# Cattle

**Published:** 1883-01

**Authors:** 


CATTLE—THE BREEDS OF, THEIR DISEASES AND TREATMENT.—The above is the title of a work nearly ready for the press, by A. S. Heath, M.D., Professor of Bovine, Ovine and Suine Pathology in the Columbia College of Comparative Medicine of New York.

The diseases and treatment of cattle, sheep and swine will be published in the form of lectures.

The aim and object of Professor Heath's work is to invite attention to the valuable breeds of foreign cattle exactly suited to the milk, meat, butter, or cheese production of the various sections of the country, resembling nearest those in which the several breeds originated. The appropriate transplanting of breeds of cattle perfected, for a special product, to a foreign location of similar climate topography, grasses, altitude, etc., seems full of thoughtful consideration and worthy of public attention.

From a perusal of the manuscript we perceive that time and expense can and must be saved to breeders and importers of stock by the valuable suggestions therein made. Reference is made to long and persistent efforts of the breeder who spent thirty years in establishing the only truly American breed of cattle, and in showing conclusively how this might have been done in less than one-quarter of the time, and at a tithe of the expense.

Great stress is laid on the value of establishing herds of grades, adapted to the several cattle industries of our great agricultural resources and localities, by the selection of pure bred males to breed to our best cows. The Doctor shows in his book that by such judicious selection and breeding, that the value of the cattle of the United States can be increared fully one-third, in a few years, while the increase by decades, formerly shows, relatively, more increase in numbers, than in the individual increase of values. He also shows plainly to us, that the most rational and profitable mode of breeding is to breed for a special product, and with reference to locality and its peculiarities, capabilities and demands.

The manuscript also states that the *world* has sixty-five valuable breeds of cattle, of which we have as yet appropriated only eleven, and that even of this sixth we have, most of them we possess so sparingly, that we can not supply one-tenth part of the males required from which to breed grades. That, with the exception of the Shorthorns and Jerseys, we have no considerable herds of these eleven pure breeds of cattle. And that of these two breeds referred to, we do not possess one of of each, where we ought to have hundreds. The book contains a chapter on American *grades*, full of most valuable suggestions and thoughtful considerations.

We have only space to speak of another most interesting feature of this coming work. And that is, the illustration of these numerous breeds of cattle, by portraits of the characteristic animals of each breed. This, it seems to us, one of the best methods of conveying valuable information, though expensive to the publisher.

